# The synergistic effect of the triglyceride-glucose index and serum uric acid on the prediction of major adverse cardiovascular events after coronary artery bypass grafting: a multicenter retrospective cohort study

**DOI:** 10.1186/s12933-023-01838-z

**Published:** 2023-05-02

**Authors:** Zhenguo Wu, Cheng Cheng, Xiangfei Sun, Juan Wang, Dachuan Guo, Sha Chen, Yerui Zhang, Xiaoyu Liu, Li Liu, Cheng Zhang, Jianmin Yang

**Affiliations:** 1 National Key Laboratory for Innovation and Transformation of Luobing Theory; The Key Laboratory of Cardiovascular Remodeling and Function Research, Chinese Ministry of Education, Chinese National Health Commission and Chinese Academy of Medical Sciences; Department of Cardiology, Qilu Hospital of Shandong University, Jinan, Shandong China; 2 Department of Cardiology, Shengjing Hospital of China Medical University, Shenyang, 110004 Liaoning China; 3 Department of Cardiovascular Surgery, Shandong Provincial Hospital, Cheeloo College of Medicine, Shandong University, Jinan, Shandong China; 4 Department of Cardiovascular Surgery, Shandong Provincial Hospital Affiliated to Shandong First Medical University, Jinan, Shandong China; 5 Department of Cardiology, The Second Hospital of Shandong University, Jinan, Shandong China

**Keywords:** Triglyceride-glucose index, Insulin resistance, Uric acid, Hyperuricemia, Coronary artery bypass grafting, Cardiovascular risk, Interaction analysis

## Abstract

**Background:**

Elevated serum uric acid (SUA) is regarded as a risk factor for the development of cardiovascular diseases. Triglyceride-glucose (TyG) index, a novel surrogate for insulin resistance (IR), has been proven to be an independent predictor for adverse cardiac events. However, no study has specifically focused on the interaction between the two metabolic risk factors. Whether combining the TyG index and SUA could achieve more accurate prognostic prediction in patients undergoing coronary artery bypass grafting (CABG) remains unknown.

**Methods:**

This was a multicenter, retrospective cohort study. A total of 1225 patients who underwent CABG were included in the final analysis. The patients were grouped based on the cut-off value of the TyG index and the sex-specific criteria of hyperuricemia (HUA). Cox regression analysis was conducted. The interaction between the TyG index and SUA was estimated using relative excess risk due to interaction (RERI), attributable proportion (AP), and synergy index (SI). The improvement of model performance yielded by the inclusion of the TyG index and SUA was examined by C-statistics, net reclassification improvement (NRI) and integrated discrimination improvement (IDI). The goodness-of-fit of models was evaluated using the Akaike information criterion (AIC), Bayesian information criterion (BIC) and χ^2^ likelihood ratio test.

**Results:**

During follow-up, 263 patients developed major adverse cardiovascular events (MACE). The independent and joint associations of the TyG index and SUA with adverse events were significant. Patients with higher TyG index and HUA were at higher risk of MACE (Kaplan–Meier analysis: log-rank *P* < 0.001; Cox regression: HR = 4.10; 95% CI 2.80–6.00, *P* < 0.001). A significant synergistic interaction was found between the TyG index and SUA [RERI (95% CI): 1.83 (0.32–3.34), *P* = 0.017; AP (95% CI): 0.41 (0.17–0.66), *P* = 0.001; SI (95% CI): 2.13 (1.13–4.00), *P* = 0.019]. The addition of the TyG index and SUA yielded a significant improvement in prognostic prediction and model fit [change in C-statistic: 0.038, *P* < 0.001; continuous NRI (95% CI): 0.336 (0.201–0.471), *P* < 0.001; IDI (95% CI): 0.031 (0.019–0.044), *P* < 0.001; AIC: 3534.29; BIC: 3616.45; likelihood ratio test: *P* < 0.001).

**Conclusions:**

The TyG index interacts synergistically with SUA to increase the risk of MACE in patients undergoing CABG, which emphasizes the need to use both measures concurrently when assessing cardiovascular risk.

**Supplementary Information:**

The online version contains supplementary material available at 10.1186/s12933-023-01838-z.

## Background

Coronary heart disease (CHD) is the leading cause of death, constituting an increasing public health burden worldwide [1, 2]. Coronary artery bypass grafting (CABG) can effectively recover the myocardial blood supply of patients with CHD and is preferred for those with multivessel disease [3, 4]. Despite advances in surgical techniques, the long-term prognosis of patients after CABG remains poor due to the complex nature of coronary lesions [5, 6]. Therefore, it is crucial to identify reliable prognostic factors for patients who underwent CABG.

Previous studies have shown that patients with diabetes mellitus (DM) derive more benefit from CABG than those without DM [7–9], and as a result, patients selected for CABG tend to suffer from more metabolic risk factors. Insulin resistance (IR) and hyperuricemia (HUA) are both important metabolic risk factors. They promote each other through multiple mechanisms and ultimately promote the progression of atherosclerosis [10–12].

The triglyceride-glucose (TyG) index has recently been regarded as a reliable indicator of IR, which is more economical and convenient than traditional assessment methods and shows a high degree of consistency with the hyperinsulinemic-euglycemic clamp [13–15]. Among patients after revascularization, those with high levels of TyG index usually had poor prognosis [15–17]. Chen et al. found that the TyG index was an independent prognostic factor in diabetic patients after CABG [18].

Serum uric acid (SUA) homeostasis depends on its production, excretion and reabsorption [19]. Multiple factors can disrupt the homeostasis of SUA and lead to HUA. There is growing evidence that HUA is associated with higher risk of CHD morbidity and mortality [20–22].

However, the prognostic value of the TyG index and SUA in patients after CABG remains unclear so far. Moreover, no study has focused on the synergistic effect of the TyG index and SUA on the prediction of adverse cardiovascular events after CABG. In the present study, we sought to investigate the prognostic value of the TyG index and SUA in patients undergoing CABG and further evaluated the synergistic effect of the two indicators.

## Methods

### Study population

The study was approved by the Ethics Review Committee of Shandong Provincial Hospital, The Second Hospital of Shandong University and Qilu Hospital of Shandong University and was carried out in compliance with the Helsinki Declaration. The patients provided verbal informed consent allowing the retrospective use of their anonymized data for health-related research, which was allowed by the Ethics Committee.

**Fig. 1:**
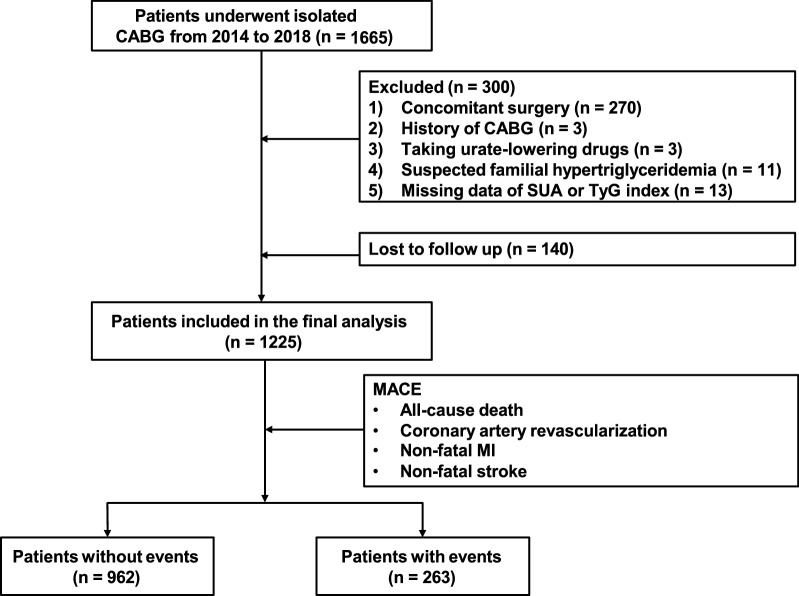
Flow diagram of patient selection. *CABG* coronary artery bypass grafting, *MACE* major adverse cardiovascular events, *MI* myocardial infarction

This multicenter retrospective cohort study included past-CABG patients from 3 tertiary public hospitals (Shandong Provincial Hospital, The Second Hospital of Shandong University and Qilu Hospital of Shandong University). From June 2014 to June 2018, 1665 consecutive patients who underwent CABG were screened. Among them, 270 were excluded due to concomitant surgery (valve surgery, surgical ablation or congenital heart surgery), 3 were excluded because of the history of CABG, 3 were excluded because of taking urate-lowering drugs, 11 were excluded because of suspected familial hypertriglyceridemia (triglyceride ≥ 5.65 mmol/L), and 13 were excluded due to missing data of SUA or data for TyG index calculation. A total of 1365 patients met all inclusion criteria and were followed up by telephone from July 2022 to November 2022. Finally, 1225 (89.7%) patients completed the telephone survey and were enrolled for the final analysis (Fig. 1).

### Data collection

Data on demographic characteristics, medical history, personal history and medication usage were collected through the electronic medical records system. Venous blood samples were drawn after overnight fasting and the levels of fasting plasma glucose (FPG), SUA, serum creatinine (SCr), and lipid profile were measured. Hypertension was diagnosed when systolic blood pressure ≥ 140 mmHg and/or diastolic blood pressure ≥ 90 mmHg, or if patients were on blood pressure-lowering therapies. Patients were classified as having diabetes mellitus (DM) if their casual blood glucose ≥ 11.1 mmol/L, or FPG ≥ 7.0 mmol/L, or if they were taking hypoglycemic drugs. Hyperlipidemia was defined based on ICD-10 code E78 with lipid-lowering drugs or total cholesterol (TC) ≥ 240 mg/dL [23]. The diagnosis of HUA was based on the sex-specific criteria: SUA level above 7 mg/dL in male and above 6 mg/dL in female [24]. Patients with a self-reported previous diagnosis of hypertension, DM, hyperlipidemia or HUA, which was confirmed by corresponding medical records, were also identified as having hypertension, DM, hyperlipidemia or HUA. Patients having 2-vessel or 3-vessel disease were classified as having multivessel disease and those with ≥ 50% stenosis occurring in the left main coronary artery were identified as containing the left main disease. Family history of coronary heart disease (FH-CHD) was defined as premature CHD in the immediate family (male < 55 or female < 65 years), which was determined by patient query. The eGFR was calculated using the following formula: eGFR (mL/min/1.73 m^2^) = 175 × SCr (mg/dL)^−1.234^ × age (year)^−0.179^ × 0.79 (if female) [25]. The TyG index was calculated based on fasting triglyceride (TG) and FPG: Ln [TG (mg/dL) × FPG (mg/dL)/2] [26].

### Outcome

The first occurrence of a major adverse cardiovascular event (MACE), including all-cause death, non-fatal myocardial infarction (MI), non-fatal stroke or repeat coronary artery revascularization, was chosen as the primary endpoint in the present study. All-cause death was defined as death from cardiac or non-cardiac causes and only the death information during the follow-up was collected. Early in-hospital mortality was excluded from the analysis because of the known differences between early and late death hazard functions following CABG [27]. Non-fatal MI was confirmed using WHO criteria: typical symptoms plus electrocardiographic changes or elevated heart enzymes [28]. Repeat coronary artery revascularization included any ischemia-driven revascularization. We adopted the definition of ischemia-driven revascularization set by the EXCEL Trial [29]. All-cause death and cardiac death were analyzed as the secondary endpoints.

### Statistical analysis

Statistical analysis was performed with SPSS version 25.0 (SPSS, Chicago, IL, United States) and R software version 4.1.3 (R Foundation for Statistical Computing, Vienna, Austria). Differences were considered significant at *P* < 0.05. We initially performed receiver operating characteristic (ROC) curve analysis to determine the optimal cut-off value of the TyG index (the cut-off value of the TyG index for predicting MACE = 8.87). The baseline characteristics of the patients were described and compared based on the cut-off value of the TyG index and the presence or absence of HUA. Continuous variables were reported as mean ± standard deviation (SD) or median (interquartile range) and analyzed by the Student’s t-test or the Mann–Whitney U test, as appropriate. Number (percentage) and chi-square tests were used to describe and compare the categorical variables.

The Kaplan–Meier method was used to generate cumulative event curves, which were stratified by the cut-off value of the TyG index and HUA, respectively. The survival difference between groups was assessed by the log-rank test. Three Cox proportional hazards regression models were built to evaluate the independent association of the TyG index and SUA with the primary endpoint. We adjusted for age and gender in Model 1. The variables with *P* < 0.05 in univariate analysis entered Model 2. Variables that showed a univariate relationship with adverse events or potentially associated with clinical outcome were controlled in the fully adjusted model (Model 3), including variables in Model 2 plus gender, body mass index (BMI), drinking, hyperlipidemia, duration of surgery, coronary artery bypass grafting (OPCABG), number of grafts, use of arterial grafts, antiplatelet drugs, statins, hypoglycemic drugs and urate-lowering drugs. First, the TyG index and SUA were included together in the models as continuous variables. To limit the influence of extreme observations, the two variables of interest were further standardized to z score, which indicates the effect size per SD increase. The two variables were also included in the models as categorical variables, based on the cut-off value of the TyG index and the sex-specific criteria of HUA. We performed a collinearity diagnosis, showing that the degree of collinearity among variables was acceptable [all variance inflation factors (VIF) < 5] [30].

To investigate the joint predictive value of the TyG index and SUA on MACE, we divided the patients into four groups: Group 1: TyG ≤ 8.87 and Non-HUA, Group 2: TyG > 8.87 and Non-HUA, Group 3: TyG ≤ 8.87 and HUA, and Group 4: TyG > 8.87 and HUA. Corresponding Kaplan–Meier curves were generated followed by the log-rank test. The joint predictive value was further analyzed via the fully adjusted Cox regression model. To test the interaction between the TyG index and SUA, we calculated relative excess risk due to interaction (RERI), attributable proportion (AP), and synergy index (SI), as previously documented [31–33]. The incremental predictive value yielded by the inclusion of the TyG index or SUA was examined using C-statistics, net reclassification improvement (NRI) and integrated discrimination improvement (IDI). The goodness-of-fit of models was evaluated using the Akaike information criterion (AIC) and Bayesian information criterion (BIC) and the χ^2^ likelihood ratio test was performed.

## Results

### Main characteristic of participants

The ROC curve analysis showed that the optimal cut-off value of the TyG index for predicting MACE was 8.87, based on the maximum value of the Youden Index (Additional file 1: Table S1). Table 1 reported the general characteristics of the study population grouped by the cut-off value of the TyG index. In total, the study cohort included 1225 patients and male patients accounted for 70.0% (n = 857). Patients whose TyG index above the cut-off value were younger, tend to be female, and showed higher levels of BMI, FPG, TC, TG, low-density lipoprotein cholesterol (LDL-C), SUA and a lower level of high-density lipoprotein cholesterol (HDL-C). In patients with high TyG index, there were more individuals who had multivessel disease, DM, hypertension and hyperlipidemia, and were treated with hypoglycemic drugs and fibrates. Meanwhile, more patients underwent OPCABG in the high TyG index group. Moreover, the incidence of MACE was higher in patients with the TyG index above the cut-off value (Table 1).

**Table 1:** Baseline characteristics of the study population according to the TyG index

Variables	All (n = 1225)	TyG ≤ 8.87 (n = 774)	TyG > 8.87 (n = 451)	*P*-value
General conditions				
Age (years)	62.79 ± 8.18	63.16 ± 8.22	62.12 ± 8.08	**0.032**
Male, n (%)	857 (70.0)	569 (73.5)	288 (63.9)	**< 0.001**
BMI (kg/m^2^)	25.67 ± 3.58	25.42 ± 3.64	26.11 ± 3.43	**0.001**
LVEF (%)	58.12 ± 10.52	58.46 ± 10.30	57.54 ± 10.88	0.142
Previous MI, n (%)	259 (21.1)	154 (19.9)	105 (23.3)	0.162
Previous stroke, n (%)	185 (15.1)	104 (13.4)	81 (18.0)	**0.033**
Previous PCI, n (%)	122 (10.0)	80 (10.3)	42 (9.3)	0.564
Left main disease, n (%)	282 (23.0)	171 (22.1)	111 (24.6)	0.312
Multivessel disease, n (%)	1147 (93.6)	710 (91.7)	437 (96.9)	**< 0.001**
Risk factors, n (%)				
Current smoking	339 (27.7)	207 (26.7)	132 (29.3)	0.341
Current drinking	316 (25.8)	199 (25.7)	117 (25.9)	0.929
FH-CHD	247 (20.2)	145 (18.7)	102 (22.6)	0.102
DM	395 (32.2)	164 (21.2)	231 (51.2)	**< 0.001**
Hypertension	767 (62.6)	451 (58.3)	316 (70.1)	**< 0.001**
Hyperlipidemia	394 (32.2)	215 (27.8)	179 (39.7)	**< 0.001**
Surgical procedure				
Duration of surgery (min)	270.00 (240.00–310.00)	270.00 (240.00–306.25)	270.00 (240.00–315.00)	0.425
OPCABG, n (%)	139 (11.3)	75 (9.7)	64 (14.2)	**0.017**
Number of grafts	3.60 ± 0.98	3.56 ± 0.99	3.65 ± 0.96	0.134
Use of arterial grafts, n (%)	1171 (95.6)	738 (95.3)	433 (96.0)	0.587
Laboratory tests				
FPG (mmol/L)	5.41 (4.77–6.84)	4.99 (4.56–5.66)	6.97 (5.57–9.20)	**< 0.001**
TC (mmol/L)	4.10 (3.48–4.96)	3.90 (3.30–4.62)	4.56 (3.78–5.18)	**< 0.001**
TG (mmol/L)	1.32 (0.99–1.77)	1.08 (0.86–1.34)	1.95 (1.56–2.60)	**< 0.001**
LDL-C (mmol/L)	2.45 (1.92–3.04)	2.36 (1.85–2.86)	2.66 (2.13–3.34)	**< 0.001**
HDL-C (mmol/L)	1.12 ± 0.26	1.14 ± 0.27	1.08 ± 0.24	**< 0.001**
eGFR (mL/min/1.73 m^2^)	107.38 ± 29.59	107.42 ± 31.04	107.40 ± 26.97	0.990
SUA (μmol/L)	307.00 (258.50–372.00)	304.00 (257.00–363.00)	317.00 (262.00–383.00)	**0.013**
Medications at the time of discharge, n (%)				
Antiplatelet drugs	1214 (99.1)	768 (99.2)	446 (98.9)	0.777
Statins	1023 (83.5)	652 (84.2)	371 (82.3)	0.369
Fibrates	83 (6.8)	38 (4.9)	45 (10.0)	**0.001**
Hypoglycemic drugs	285 (23.3)	117 (15.1)	168 (37.3)	**< 0.001**
Urate-lowering drugs	14 (1.1)	7 (0.9)	7 (1.6)	0.304
EuroSCORE II	1.45 (1.00–2.41)	1.43 (1.00–2.44)	1.47 (0.99–2.36)	0.822
MACE, n (%)	263 (21.5)	118 (15.2)	145 (32.2)	**< 0.001**

*TyG index* triglyceride-glucose index, *BMI* body mass index, *LVEF* left ventricle ejection fraction, *MI* myocardial infarction, *PCI* percutaneous coronary intervention, *FH-CHD* family history of coronary heart disease, *DM* diabetes mellitus, *OPCABG* off-pump coronary artery bypass grafting, *FPG* fasting plasma glucose, *TC* total cholesterol, *TG* triglyceride, *LDL-C* low-density lipoprotein-cholesterol, *HDL-C* high-density lipoprotein-cholesterol, *eGFR* estimated glomerular filtration rate, *SUA* serum uric acid, *EuroSCORE* European System for Cardiac Operative Risk Evaluation score, *MACE* major adverse cardiovascular event

The participants were further grouped into HUA group and non-HUA group, as shown in Table 2. Compared to non-HUA individuals, those with HUA showed higher levels of TG, LDL-C and SUA and lower levels of HDL-C and eGFR. Compared with non-HUA patients, those with HUA tend to have more MACE (Table 2).

**Table 2:** Baseline characteristics of the study population according to SUA

Variables	Non-HUA (n = 1030)	HUA (n = 195)	*P*-value
General conditions			
Age (years)	62.88 ± 8.11	62.24 ± 8.58	0.315
Male, n (%)	731 (71.0)	126 (64.6)	0.076
BMI (kg/m^2^)	25.59 ± 3.54	26.08 ± 3.78	0.086
LVEF (%)	58.35 ± 10.26	56.90 ± 11.76	0.109
Previous MI, n (%)	215 (20.9)	44 (22.6)	0.596
Previous stroke, n (%)	160 (15.5)	25 (12.8)	0.332
Previous PCI, n (%)	108 (10.5)	14 (7.2)	0.157
Left main disease, n (%)	238 (23.1)	44 (22.6)	0.869
Multivessel disease, n (%)	968 (94.0)	179 (91.8)	0.252
Risk factors, n (%)			
Current smoking	288 (28.0)	51 (26.2)	0.605
Current drinking	269 (26.1)	47 (24.1)	0.556
FH-CHD	215 (20.9)	32 (16.4)	0.154
DM	331 (32.1)	64 (32.8)	0.851
Hypertension	634 (61.6)	133 (68.2)	0.078
Hyperlipidemia	333 (32.3)	61 (31.3)	0.774
Surgical procedure			
Duration of surgery (min)	270.00 (240.00–310.00)	270.00 (230.00–310.00)	0.566
OPCABG, n (%)	121 (11.7)	18 (9.2)	0.310
Number of grafts	3.61 ± 0.99	3.55 ± 0.97	0.457
Use of arterial grafts, n (%)	985 (95.6)	186 (95.4)	0.878
Laboratory tests			
FPG (mmol/L)	5.40 (4.75–6.81)	5.60 (4.83–6.98)	0.144
TC (mmol/L)	4.07 (3.48–4.92)	4.32 (3.51–5.08)	0.112
TG (mmol/L)	1.28 (0.97–1.72)	1.50 (1.16–2.13)	**< 0.001**
LDL-C (mmol/L)	2.44 (1.90–2.98)	2.48 (2.04–3.31)	**0.019**
HDL-C (mmol/L)	1.13 ± 0.25	1.08 ± 0.29	**0.044**
eGFR (mL/min/1.73 m^2^)	109.73 ± 29.16	95.18 ± 28.91	**< 0.001**
SUA (μmol/L)	291.00 (249.75–340.00)	454.00 (426.00–492.00)	**< 0.001**
Medications at the time of discharge, n (%)			
Antiplatelet drugs	1019 (98.9)	195 (100.00)	0.300
Statins	856 (83.1)	167 (85.6)	0.382
Fibrates	68 (6.6)	15 (7.7)	0.579
Hypoglycemic drugs	241 (23.4)	44 (22.6)	0.800
Urate-lowering drugs	0	14 (7.2)	**< 0.001**
EuroSCORE II	1.43 (0.99–2.40)	1.52 (1.04–2.62)	0.369
MACE, n (%)	196 (19.0)	67 (34.4)	**< 0.001**

*SUA* serum uric acid, *HUA* hyperuricemia, *BMI* body mass index, *LVEF* left ventricle ejection fraction, *MI* myocardial infarction, *PCI* percutaneous coronary intervention, *FH-CHD* family history of coronary heart disease, *DM* diabetes mellitus, *OPCABG* off-pump coronary artery bypass grafting, *FPG* fasting plasma glucose, *TC* total cholesterol, *TG* triglyceride, *LDL-C* low-density lipoprotein-cholesterol, *HDL-C* high-density lipoprotein-cholesterol, *eGFR* estimated glomerular filtration rate, *SUA* serum uric acid, *EuroSCORE* European System for Cardiac Operative Risk Evaluation score, *MACE* major adverse cardiovascular event

### Respective predictive value of the TyG index and SUA for MACE

**Fig. 2:**
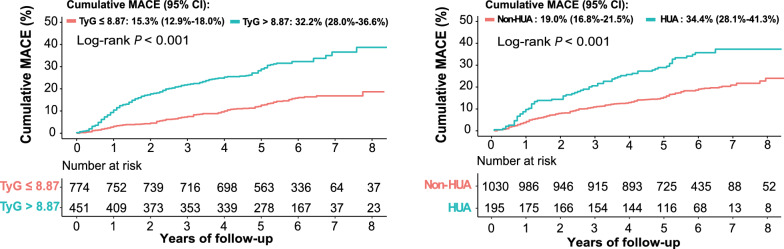
Kaplan–Meier survival curves according to the TyG index and HUA. *TyG index* triglyceride-glucose index, *HUA* hyperuricemia, *MACE* major adverse cardiovascular events

In our study cohort (median follow-up time: 69 months, interquartile range: 56–76 months), MACE occurred in 263 (21.5%) patients (median time from index CABG to event: 28 months, interquartile range: 12–50 months). The first occurrence of MACE included 82 (6.7%) all-cause death [53 died from cardiac causes and 29 died from other causes], 70 (5.7%) non-fatal MI, 66 (5.4%) non-fatal stroke and 45 (3.7%) repeat revascularization. Kaplan–Meier survival curves were drawn according to the cut-off value of the TyG index and HUA (Fig. 2). Cumulative rate of MACE was significantly higher in patients with the TyG index above the cut-off value (log-rank test, *P* < 0.001). Meanwhile, compared to patients without HUA, those with HUA exhibited a higher MACE risk (log-rank test, *P* < 0.001).

Univariate Cox regression analysis showed that the TyG index and SUA were significantly associated with MACE (Additional file 1: Table S2). The predictive value of the TyG index and SUA remained significant after adjusting for other cardiovascular risk factors (Table 3). Compared to patients with low levels of TyG index and SUA, the risk of MACE was 2.18 times higher for individuals with a high TyG index and 1.84 times higher for individuals with HUA.

**Table 3:** Multivariate Cox regression analysis for MACE

Variables	HR (95% CI)		
	Model 1	Model 2	Model 3
TyG index			
Per unit increase	1.70 (1.43–2.04)***	1.49 (1.22–1.82)***	1.53 (1.24–1.89)***
Per SD increase	1.40 (1.25–1.56)***	1.28 (1.13–1.45)***	1.30 (1.14–1.49)***
TyG ≤ 8.87	1 (Reference)	1 (Reference)	1 (Reference)
TyG > 8.87	2.42 (1.89–3.10)***	2.14 (1.64–2.79)***	2.18 (1.66–2.86)***
SUA			
Per unit increase	1.00 (1.00–1.01)***	1.00 (1.00–1.01)***	1.00 (1.00–1.01)***
Per SD increase	1.35 (1.20–1.51)***	1.34 (1.19–1.52)***	1.35 (1.18–1.55)***
Non-HUA	1 (Reference)	1 (Reference)	1 (Reference)
HUA	1.90 (1.43–2.51)***	1.87 (1.40–2.50)***	1.84 (1.37–2.49)***

Model 1: Adjusted for age and gender

Model 2: Adjusted for variables with *P*-value < 0.05 in univariate analysis, including age, LVEF, left main disease, smoking, DM, hypertension, TC, LDL-C and eGFR

Model 3: Adjusted for all the variables in Model 2 plus gender, BMI, drinking, hyperlipidemia, duration of surgery, OPCABG, number of grafts, use of arterial grafts, antiplatelet drugs, statins, hypoglycemic drugs and urate-lowering drugs

*TyG index* triglyceride-glucose index, *SUA* serum uric acid, *MACE* major adverse cardiovascular events, *HUA* hyperuricemia

***P < 0.001

### Joint influences of the TyG index and SUA on incident MACE risk

**Fig. 3:**
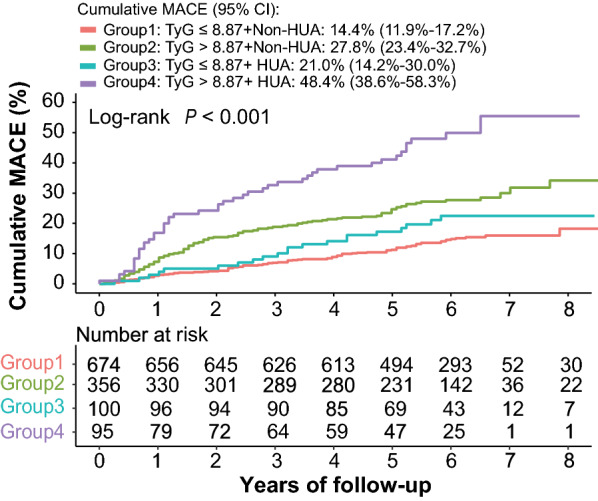
Kaplan–Meier survival curves across TyG index and HUA groups. *TyG index* triglyceride-glucose index, *HUA* hyperuricemia, *MACE* major adverse cardiovascular events

Patients were divided into four groups according to the cut-off value of the TyG index and the sex-specific criteria of HUA. Kaplan–Meier analysis was performed to compare the risk of MACE among the four groups (Fig. 3). Results showed that the risk of incident MACE was highest in patients with TyG index > 8.87 and HUA, and the cumulative rate of MACE was significantly lower in individuals with TyG index below the cut-off value and without HUA (log-rank test, *P* < 0.001).

The combined influences of the TyG index and SUA on MACE were further verified by Cox regression analysis. The highest risk of MACE was found among patients with TyG index > 8.87 and HUA (HR = 4.46; 95% CI 3.13–6.33, *P* < 0.001). The result remained significant in multivariable-adjusted analysis. The adjusted HR for MACE was 4.10 (95% CI 2.80–6.00) in patients with TyG index above the cut-off value and HUA, compared to individuals in Group 1 (Table 4). Other statistically significant variables included age, left ventricle ejection fraction (LVEF), left main disease, smoking, hypertension and TC (Additional file 1: Table S3).

**Table 4:** Joint association of TyG index and SUA with MACE

	Univariate regression		Multivariate regression^a^	
	HR (95% CI)	*P* value	HR (95% CI)	*P* value
TyG ≤ 8.87 and non-HUA	1 (Reference)		1 (Reference)	
TyG > 8.87 and non-HUA	2.12 (1.61–2.81)	**< 0.001**	2.11 (1.55–2.86)	**< 0.001**
TyG ≤ 8.87 and HUA	1.50 (0.93–2.40)	0.094	1.67 (1.02–2.73)	**0.041**
TyG > 8.87 and HUA	4.46 (3.13–6.33)	**< 0.001**	4.10 (2.80–6.00)	**< 0.001**

*TyG index* triglyceride-glucose index, *SUA* serum uric acid, *MACE* major adverse cardiovascular events, *HUA* hyperuricemia

*P* values in bold are < 0.05

^a^Adjusted for age, gender, BMI, LVEF, left main disease, current smoking, current drinking, DM, hypertension, hyperlipidemia, duration of surgery, OPCABG, number of grafts, use of arterial grafts,TC, LDL-C, eGFR, antiplatelet drugs, statins, hypoglycemic drugs and urate-lowering drugs

We then investigated the joint impacts of the TyG index and SUA on all-cause death and cardiac death. The highest and statistically significant risk was observed among patients in Group 4 [HR (95% CI) of all-cause death: 5.10 (2.66–9.79), *P* < 0.001; HR (95% CI) of cardiac death: 6.07 (2.66–13.85), *P* < 0.001)] (Additional file 1: Table S4).

Sensitivity analysis was performed after exclusion of patients with renal insufficiency or individuals receiving lipid-lowering or hypoglycemic treatment at admission, which showed that our results were robust (Additional file 1: Table S5).

### Subgroup analysis

**Fig. 4:**
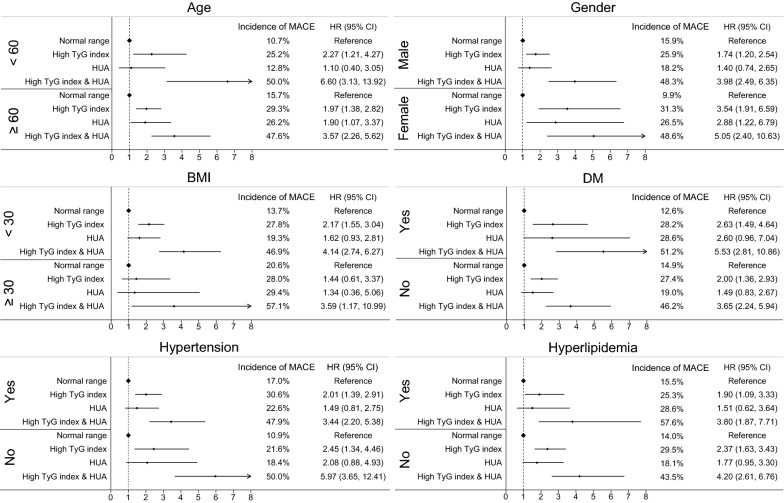
Subgroup analysis. *TyG index* triglyceride-glucose index, *HUA* hyperuricemia, *BMI* body mass index, *DM* diabetes mellitus

Subgroup analysis was performed based on age, gender, BMI, DM, hypertension and hyperlipidemia. Figure 4 showed the combined association of the TyG index and SUA with MACE among subgroups. Generally, patients who had both high TyG index and HUA were more significantly associated with adverse cardiovascular outcomes, no matter in which subgroup (Fig. 4).

### Interaction between the TyG index and SUA

The interaction analysis showed that the combined effect of the TyG index and HUA was significantly greater than the sum of their individual effects and the risk of MACE increased by 41% attributed to the interaction (Table 5). This synergistic interaction seemed to be more prominent in patients without DM and obesity (Additional file 1: Table S6).

**Table 5:** Synergistic interaction between the TyG index and SUA

	Value	95% CI	*P*-value
RERI	1.83	0.32–3.34	**0.017**
AP	0.41	0.17–0.66	**0.001**
SI	2.13	1.13–4.00	**0.019**

*TyG index* triglyceride-glucose index, *SUA* serum uric acid, *RERI* relative excess risk due to interaction, *AP* attributable proportion, *SI* synergy index

*P* values in bold are < 0.05

### The incremental predictive value of the TyG index and SUA

According to the C-statistic, continuous NRI and IDI, the addition of either the TyG index or SUA to the baseline model yielded a moderate but significant improvement in outcome prediction. We further added both the TyG index and SUA together to the baseline model, which achieved the greatest improvement of the model performance and showed a reduction of both false positives and false negatives [event NRI (95% CI): 0.141 (0.021–0.260), *P* = 0.021; non-event NRI (95% CI): 0.195 (0.134–0.257), *P* < 0.001] (Table 6).

**Table 6:** The incremental predictive value of the TyG index and SUA for MACE

	C-Statistic (95% CI)	*P*-value	Continuous NRI (95% CI)	*P*-value	IDI (95% CI)	*P*-value
Model 3 without TyG and SUA	0.658 (0.625–0.691)	**< 0.001**	Reference		Reference	
Model 3 + TyG	0.684 (0.651–0.717)	**< 0.001**	0.299 (0.165–0.434)	**< 0.001**	0.014 (0.006–0.022)	**< 0.001**
Model 3 + SUA	0.677 (0.644–0.710)	**< 0.001**	0.266 (0.130–0.401)	**< 0.001**	0.022 (0.012–0.032)	**< 0.001**
Model 3 + TyG + SUA	0.696 (0.663–0.729)	**< 0.001**	0.336 (0.201–0.471)	**< 0.001**	0.031 (0.019–0.044)	**< 0.001**

*TyG index* triglyceride-glucose index, *SUA* serum uric acid, *MACE* major adverse cardiovascular events, *NRI* net reclassification improvement, *IDI* integrated discrimination improvement

*P* values in bold are < 0.05

### Assessment of model goodness-of-fit

As shown in Table 7, the addition of either the TyG index or SUA to the baseline model improved model fit significantly. The model that included both the TyG index and SUA was the best-fit model, with the lowest AIC and BIC values and the likelihood ratio test was significant (Table 7).

**Table 7:** Assessment of the goodness-of-fit of models

	Model 3 without TyG and SUA	Model 3 + TyG	Model 3 + SUA	Model 3 + TyG + SUA
AIC	3570.26	3550.77	3547.55	3534.29
BIC	3645.28	3629.36	3626.13	3616.45
χ^2^	Reference	21.49	24.72	39.97
df	Reference	1	1	2
*P-*value	Reference	**< 0.001**	**< 0.001**	**< 0.001**

*TyG index* triglyceride-glucose index, *SUA* serum uric acid, *AIC* Akaike information criterion, *BIC* Bayesian information criterion

*P* values in bold are < 0.05

## Discussion

Based on our analysis, we have the following main findings: (1) Both the TyG index and SUA were independent prognostic factors for post-CABG patients. (2) Patients who had concurrent high TyG index and HUA had the greatest risk of MACE compared to those with neither risk factor elevated. (3) The TyG index and SUA synergistically increased the risk of incident MACE in patients after CABG.

Several studies have investigated the relationship between IR and adverse cardiovascular events after revascularization [34–36]. In this regard, the TyG index exhibited great potential as a cardiovascular risk predictor [15–17, 37, 38]. In the present study, we confirmed again that the TyG index was independently associated with MACE in patients who underwent CABG, consistent with previous findings in patients with DM [18, 39].

Whether the SUA is an independent risk factor for adverse cardiovascular events is still controversial [40]. Studies in animal models have proposed potential mechanisms leading to cardiovascular events in response to HUA [41, 42]. Several epidemiological studies have also revealed the association between SUA and cardiovascular risk [43–45]. However, no independent predictive value of SUA was found in a Mendelian randomization study [46]. In our present study, we found that SUA was associated with MACE in patients after CABG, which was independent of traditional cardiovascular risk factors.

To the best of our knowledge, the current study provided the first proof of the synergistic effect between the TyG index and SUA in increasing the risk of MACE. We use the additive model to perform interaction analysis, which is suitable for biological and etiological investigations [47, 48]. Despite the precise mechanism of synergistic interaction remaining unclear, some previous studies provided valuable mechanistic insights. IR could reduce UA excretion and enhance UA reabsorption [10, 49]. Meanwhile, HUA could adversely interfere with glucose uptake in skeletal muscle and induce oxidative changes in adipocytes, and lead to IR [50–52]. They promote each other and ultimately promote the progression of atherosclerosis through inflammation, oxidative stress, endothelial dysfunction and renin–angiotensin–aldosterone system activation [53–56]. Moreover, CATAMERI Study also provided evidence of interaction between IR and SUA in promoting vascular damage [57].

The ability of the TyG index to improve model performance was found in previous studies [58, 59], but the usefulness of the TyG index in the improvement of risk prediction after CABG was uncertain. In our current data analysis, NRI was used to measure the clinically meaningful improvement in risk classification, and IDI was used to represent the improvement in risk discrimination. The present study discovered the incremental predictive value of the TyG index and SUA in post-CABG patients for the first time. Further analysis revealed that combining the two factors could yield the greatest improvement in risk classification and discrimination and model fit. The TyG index and SUA could be used together for post-CABG risk forecasting and stratification. In addition, improvement was seen in the C-statistics after adding the TyG index and SUA, which was often very difficult to achieve in models with strong predictive power. In the current study, non-inclusion of several variables that were already known to determine post-CABG outcome made the base model weak. This made it easier to improve the C-statistics.

To rule out the influence of lipid-lowering or hypoglycemic treatment on the calculation of the TyG index, data were reanalyzed after excluding patients taking lipid-lowering or hypoglycemic drugs. We further excluded individuals with renal insufficiency, which can substantially influence the SUA level. We also explored the joint association of the TyG index and SUA with MACE among different subgroups. The results did not change significantly in sensitivity analysis and subgroup analysis, indicating that the predictive value of the TyG index and SUA for MACE was applicable to almost all populations.

This study has several limitations. First, although this is a multicenter cohort study, the retrospective design and its inherent limitations cannot be avoided. Second, several variables associated with post-CABG adverse events were not included in the regression model, such as admission type, clinical syndrome, pre-operative co-morbidity and pre-operative risk. In addition, although multiple variables associated with the progression of atherosclerosis were adjusted, we did not adjust for several variables affecting graft failure and further driving the occurrence of repeat revascularization. Therefore, no statement can be made whether the association of the TyG index and SUA with MACE was independent of these risk factors. Third, repeat revascularization was difficult to assess though it was explicitly defined and confirmed by carefully reviewing the corresponding medical records. Fourth, the HbA1c levels were not measured and data for the Society of Thoracic Surgeons (STS) risk score and Syntax score calculation were not available in most patients. Finally, our study population had a low rate of therapeutic intervention for uric acid and diabetes, which limited the generalization of results to other populations. Further prospective studies among populations with a higher therapeutic intervention rate could address the limitations of this study and confirm our findings.

## Conclusion

In conclusion, the results emerging from our study revealed the prognostic value of the TyG index and SUA in patients who underwent CABG. Furthermore, our data provided novel information on the synergistic interaction between the TyG index and SUA. The combination of the TyG index and SUA could be proposed as a useful prognostic indicator in post-CABG patients.

## Supplementary Information

**Additional file 1: Table S1.** ROC curve analysis determined optimal cut-off value of the TyG index for predicting MACE. **Table S2.** Univariate Cox regression analysis for MACE. **Table S3.** HR for variables in the multivariate Cox regression analysis. **Table S4.** Joint association of TyG index and SUA with all-cause death and cardiac death. **Table S5.** Sensitivity analysis for the joint association of the TyG index and SUA with MACE. **Table S6.** Synergistic interaction between the TyG index and SUA in patients with and without DM/obesity.

## Data Availability

The datasets used and/or analyzed during the current study are available from the corresponding author on reasonable request.

## Abbreviations

SUA: Serum uric acid
TyG index: Triglyceride-glucose index
IR: Insulin resistance
CABG: Coronary artery bypass grafting
HUA: Hyperuricemia
RERI: Relative excess risk due to interaction
AP: Attributable proportion
SI: Synergy index
NRI: Net reclassification improvement
IDI: Integrated discrimination improvement
MACE: Major adverse cardiovascular events
CHD: Coronary heart disease
DM: Diabetes mellitus
FPG: Fasting plasma glucose
SCr: Serum creatinine
TC: Total cholesterol
FH-CHD: Family history of coronary heart disease
TG: Triglyceride
MI: Myocardial infarction
ROC curve: Receiver operating characteristic curve
SD: Standard deviation
BMI: Body mass index
LVEF: Left ventricular ejection fraction
OPCABG: Off-pump coronary artery bypass grafting
HDL-C: High-density lipoprotein cholesterol
LDL-C: Low-density lipoprotein cholesterol
VIF: Variance inflation factors
