# Supplementary material for: The synergistic effect of the triglyceride-glucose index and serum uric acid on the prediction of major adverse cardiovascular events after coronary artery bypass grafting: a multicenter retrospective cohort study
Source: Cardiovasc Diabetol. 2023 May 2;22:103. doi: 10.1186/s12933-023-01838-z (PMC10155424; doi:10.1186/s12933-023-01838-z)
Supplement: Supplementary file 1 — Additional file 1: Table S1. ROC curve analysis determined optimal cut-off value of the TyG index for predicting MACE. Table S2. Univariate Cox regression analysis for MACE. Table S3. HR for variables in the multivariate Cox regression analysis. Table S4. Joint association of TyG index and SUA with all-cause death and cardiac death. Table S5. Sensitivity analysis for the joint association of the TyG index and SUA with MACE. Table S6. Synergistic interaction between the TyG index and SUA in patients with and without DM/obesity. [file 12933_2023_1838_MOESM1_ESM.docx]

**Table S1 ROC curve analysis determined optimal cut-off value of the TyG index for predicting MACE**

| **Test** | **optimal cut-off value** | **Sensitivity** | **Specificity** | **AUC** |
| --- | --- | --- | --- | --- |
| **TyG index** | 8.87 | 0.551 | 0.683 | 0.631 |

ROC curve, receiver operating characteristic curve; AUC, area under the curve; TyG index, triglyceride-glucose index.

**Table S2 Univariate Cox regression analysis for MACE**

| **Variables** | **HR** | **95% CI** | ***P*-value** |
| --- | --- | --- | --- |
| Age (years) | 1.03 | 1.02-1.05 | **<0.001** |
| Male | 0.89 | 0.69-1.16 | 0.392 |
| BMI (kg/m^2^) | 1.02 | 0.99-1.06 | 0.170 |
| LVEF (%) | 0.98 | 0.97-0.99 | **0.001** |
| Previous MI | 1.03 | 0.77-1.37 | 0.865 |
| Previous stroke | 1.20 | 0.87-1.65 | 0.271 |
| Previous PCI | 0.84 | 0.55-1.29 | 0.435 |
| Left main disease | 1.42 | 1.09-1.85 | **0.010** |
| Multivessel disease | 1.50 | 0.84-2.67 | 0.172 |
| Current Smoking | 1.39 | 1.08-1.79 | **0.011** |
| Current Drinking | 1.03 | 0.79-1.35 | 0.827 |
| FH-CHD | 1.04 | 0.78-1.41 | 0.778 |
| DM | 1.36 | 1.06-1.74 | **0.017** |
| Hypertension | 1.63 | 1.24-2.13 | **< 0.001** |
| Hyperlipidemia | 1.20 | 0.93-1.55 | 0.156 |
| Duration of surgery (min) | 1.00 | 1.00-1.00 | 0.202 |
| OPCABG | 1.12 | 0.77-1.61 | 0.563 |
| Number of grafts | 0.92 | 0.81-1.04 | 0.164 |
| Use of arterial grafts | 1.11 | 0.61-2.04 | 0.725 |
| FPG (mmol/L) | 1.10 | 1.05-1.14 | **< 0.001** |
| TC (mmol/L) | 1.22 | 1.13-1.33 | **< 0.001** |
| TG (mmol/L) | 1.11 | 1.04-1.18 | **0.002** |
| LDL-C (mmol/L) | 1.16 | 1.02-1.32 | **0.025** |
| HDL-C (mmol/L) | 0.65 | 0.39-1.07 | 0.089 |
| eGFR (mL/min/1.73m^2^) | 0.99 | 0.99-1.00 | **0.015** |
| Antiplatelet drugs | 0.75 | 0.24-2.33 | 0.613 |
| Statins | 1.05 | 0.75-1.46 | 0.783 |
| Fibrates | 1.24 | 0.79-1.94 | 0.343 |
| Hypoglycemic drugs | 1.14 | 0.87-1.51 | 0.341 |
| Urate-lowering drugs | 1.99 | 0.82-4.82 | 0.128 |
| SUA (μmol/L) | 1.00 | 1.00-1.01 | **<0.001** |
| SUA (Per SD) | 1.37 | 1.22-1.53 | **<0.001** |
| TyG index | 1.71 | 1.44-2.02 | **<0.001** |
| TyG index (Per SD) | 1.40 | 1.26-1.55 | **<0.001** |

*P* values in bold are < 0.05.

MACE, major adverse cardiovascular events; HR, Hazard ratio; CI, Confidence interval; BMI, body mass index; LVEF, left ventricle ejection fraction; MI, myocardial infarction; PCI, percutaneous coronary intervention; FH-CHD, family history of coronary heart disease; DM, diabetes mellitus; OPCABG, off-pump coronary artery bypass grafting; FPG, fasting plasma glucose; TC, total cholesterol; TG, triglyceride; LDL-C, low-density lipoprotein-cholesterol; HDL-C, high-density lipoprotein-cholesterol; eGFR, estimated glomerular filtration rate; SUA, serum uric acid; TyG index, triglyceride-glucose index; SD, standard deviation.

**Table S3 HR for variables in the multivariate Cox regression analysis**

| **Variables** | **HR** | **95% CI** | ***P*-value** |
| --- | --- | --- | --- |
| Age (years) | 1.04 | 1.02-1.05 | **< 0.001** |
| Male | 1.05 | 0.77-1.43 | 0.775 |
| BMI (kg/m^2^) | 1.01 | 0.98-1.05 | 0.492 |
| LVEF (%) | 0.98 | 0.97-1.00 | **0.005** |
| Left main disease | 1.34 | 1.02-1.77 | **0.037** |
| Current Smoking | 1.62 | 1.17-2.24 | **0.003** |
| Current Drinking | 0.86 | 0.61-1.20 | 0.371 |
| DM | 1.15 | 0.80-1.64 | 0.450 |
| Hypertension | 1.45 | 1.09-1.92 | **0.010** |
| Hyperlipidemia | 0.91 | 0.69-1.20 | 0.516 |
| Duration of surgery (min) | 1.00 | 1.00-1.00 | 0.328 |
| OPCABG | 1.08 | 0.71-1.65 | 0.706 |
| Number of grafts | 0.88 | 0.73-1.03 | 0.113 |
| Use of arterial grafts | 1.44 | 0.77-2.67 | 0.254 |
| TC (mmol/L) | 1.22 | 1.10-1.36 | **< 0.001** |
| LDL-C (mmol/L) | 0.86 | 0.73-1.01 | 0.068 |
| eGFR (mL/min/1.73m^2^) | 1.00 | 1.00-1.01 | 0.707 |
| Antiplatelet drugs | 1.10 | 0.34-3.60 | 0.874 |
| Statins | 1.13 | 0.80-1.59 | 0.487 |
| Hypoglycemic drugs | 0.82 | 0.55-1.20 | 0.302 |
| Urate-lowering drugs | 1.49 | 0.58-3.80 | 0.409 |

*P* values in bold are < 0.05.

HR, Hazard ratio; CI, Confidence interval; BMI, body mass index; LVEF, left ventricle ejection fraction; DM, diabetes mellitus; OPCABG, off-pump coronary artery bypass grafting; TC, total cholesterol; LDL-C, low-density lipoprotein-cholesterol; eGFR, estimated glomerular filtration rate.

**Table S4 Joint association of TyG index and SUA with all-cause death and cardiac death**

|  | **All-cause death** | | **Cardiac death** | |
| --- | --- | --- | --- | --- |
|  | **HR (95% CI)** | ***P-*value** | **HR (95% CI)** | ***P-*value** |
| **TyG ≤ 8.87 and Non-HUA** | 1 (Reference) | | 1 (Reference) | |
| **TyG > 8.87 and Non-HUA** | 1.47 (0.80-2.70) | 0.219 | 2.38 (1.11-5.10) | **0.025** |
| **TyG ≤ 8.87 and HUA** | 2.68 (1.27-5.67) | **0.010** | 3.66 (1.41-9.51) | **0.008** |
| **TyG > 8.87 and HUA** | 5.10 (2.66-9.79) | **< 0.001** | 6.07 (2.66-13.85) | **< 0.001** |

Adjusted for age, gender, BMI, LVEF, left main disease, current smoking, current drinking, DM, hypertension, hyperlipidemia, duration of surgery, OPCABG, number of grafts, use of arterial grafts, TC, LDL-C, eGFR, antiplatelet drugs, statins, hypoglycemic drugs and urate-lowering drugs.

TyG index, triglyceride-glucose index; SUA, serum uric acid; HUA, hyperuricemia.

*P* values in bold are < 0.05.

**Table S5 Sensitivity analysis for the joint association of the TyG index and SUA with MACE**

|  | **HR (95% CI)** | |
| --- | --- | --- |
|  | **Analysis 1** | **Analysis 2** |
| **TyG ≤ 8.87 and Non-HUA** | 1 (Reference) | |
| **TyG > 8.87 and Non-HUA** | 2.09 (1.38-3.15) *** | 2.20 (1.62-3.00) *** |
| **TyG ≤ 8.87 and HUA** | 1.48 (0.79-2.77) | 1.70 (1.01-2.87) * |
| **TyG > 8.87 and HUA** | 3.49 (2.06-5.90) *** | 4.05 (2.71-6.03) *** |

Analysis 1: Excluding patients receiving lipid-lowering or hypoglycemic treatment at admission (n = 484).

Analysis 2: Excluding patients with eGFR of < 60 mL/min/1.73m^2^ (n = 30).

Adjusted for age, gender, BMI, LVEF, left main disease, current smoking, current drinking, DM, hypertension, hyperlipidemia, duration of surgery, OPCABG, number of grafts, use of arterial grafts, TC, LDL-C, eGFR, antiplatelet drugs, statins, hypoglycemic drugs and urate-lowering drugs.

TyG index, triglyceride-glucose index; SUA, serum uric acid; HUA, hyperuricemia; MACE, major adverse cardiovascular events.

* *P* < 0.05

*** *P* < 0.001

**Table S6** **Synergistic interaction between the TyG index and SUA in patients with and without DM/obesity**

|  | **RERI** | ***P*-value** | **AP** | ***P*-value** | **SI** | ***P*-value** |
| --- | --- | --- | --- | --- | --- | --- |
| **DM** | 1.49 (-1.64-4.61) | 0.351 | 0.27 (-0.23-0.77) | 0.286 | 1.50 (0.61-3.69) | 0.378 |
| **Non-DM** | 1.75 (0.24-3.26) | **0.023** | 0.43 (0.15-0.72) | **0.003** | 2.37 (1.01-5.58) | **0.048** |
| **BMI ≥ 30 (kg/m^2^)** | 1.96 (-1.38-5.30) | 0.251 | 0.48 (-0.08-1.03) | 0.093 | 2.71 (0.42-17.49) | 0.295 |
| **BMI < 30 (kg/m^2^)** | 1.82 (0.16-3.48) | **0.032** | 0.41 (0.14-0.68) | **0.003** | 2.11 (1.06-4.19) | **0.033** |

TyG index, triglyceride-glucose index; SUA, serum uric acid; DM, diabetes mellitus; BMI, body mass index.
